# Real-time evaluation of a multi-agency TB-screening event for persons experiencing homelessness in a town with a low incidence of TB in England

**DOI:** 10.1017/S0950268824000402

**Published:** 2024-04-01

**Authors:** Mona Dave, Shivan Thakrar, Helen Bagnall, Jharna Kumbang

**Affiliations:** 1 UK Field Epidemiology Training Program (UK FETP), UK Health Security Agency, London, United Kingdom; 2 Field Service Midlands Regions Directorate, UK Health Security Agency, Birmingham, UK; 3 East Midlands Health Protection Team, UK Health Security Agency, Nottingham, UK

**Keywords:** Infectious disease control, persons experiencing homelessness (PEH), real-time evaluation, screening programme, tuberculosis (TB)

## Abstract

Real-time evaluation (RTE) supports populations (e.g., persons experiencing homelessness (PEH) to engage in evaluation of health interventions who may otherwise be overlooked. The aim of this RTE was to explore the understanding of TB amongst PEH, identify barriers/facilitators to attending screening for PEH alongside suggestions for improving TB-screening events targeting PEH, who have high and complex health needs. This RTE composed of free-text structured one-to-one interviews performed immediately after screening at a single tuberculosis (TB) screening event. Handwritten forms were transcribed for thematic analysis, with codes ascribed to answers that were developed into core themes. All RTE participants (n=15) learned about the screening event on the day it was held. Key concerns amongst screening attendees included: stigma around drug use, not understanding the purpose of TB screening, lack of trusted individuals/services present, too many partner organizations involved, and language barriers. Facilitators to screening included a positive welcome to the event, a satisfactory explanation of screening tests, and sharing of results. A need for improved event promotion alongside communication of the purpose of TB screening amongst PEH was also identified. A lack of trust identified by some participants suggests the range of services present should be reconsidered for future screening events.

## Introduction

Tuberculosis (TB), a bacterial infection caused by *Mycobacterium tuberculosis*, continues to contribute to substantial global mortality and morbidity. In 2021, 10.6 million people were affected by TB and 1.6 million people died from TB worldwide [1]. In low-TB incidence countries (incidence of ≤10 per 100,000 population) [2] such as England (7.8 per 100,000 population in 2021) [2], TB transmission disproportionately affects deprived populations such as persons experiencing homelessness (PEH), migrants, and ethnic minority groups [2].

Homelessness in England is defined in the Housing Act 1996 [3]. This legal definition includes persons who have no accommodation available for them to occupy (e.g. sleeping rough) and individuals with a place to sleep that is temporary accommodation (e.g. in institutions or a shelter) [3, 4]. Those who live in insecure or unfit housing also fall under the definition [3, 4].

PEH face substantial health inequalities and have high and complex health needs [5]. PEH are expected to die over 30 years earlier than the general population [6]. PEH can be at a higher risk of exposure to and transmission of TB, especially if they seek shelter or congregate in overcrowded, poorly ventilated areas and live amongst other high-risk individuals [7]. PEH may also have an increased risk of activation of latent TB and, thereafter be, more likely to develop more severe forms of active TB, than the general UK-born population. This is a result of differential vulnerabilities such as higher rates of comorbidities within these groups and differential treatment-seeking behaviour or access to healthcare [8, 9].

From a public health perspective, further person-to-person transmission of TB can be prevented by effective contact tracing, screening, prompt diagnosis, and treatment commencement. In the United Kingdom, this is led by UK Health Security Agency (UKHSA) health protection teams (HPTs) in collaboration with other stakeholders including local National Health Service (NHS) TB teams, Integrated Care Boards (ICBs), and local authorities [10].

In this article, we describe the implementation and findings of a RTE of a multi-agency TB-screening event targeted at PEH in a town with a low incidence of TB in England (Town X) following a TB cluster investigation by UKHSA.

## Background to the targeted TB-screening event

### Background to TB in town X and cluster notification

Town X is a low, but increasing, TB incidence town in England. Regular screening of workers in several local factories in Town X had been conducted before the COVID-19 pandemic by the local TB team due to historic TB cluster investigations, with a plan to recommence these in 2023. In November 2022, the East Midlands Health Protection Team (HPT) and Field Service (FS) Midlands Team at UKHSA were notified of a fifth case of active TB linked to a factory setting in Town X. At the time of investigation, UKHSA was aware of five whole-genome sequencing (WGS) clusters of TB circulating in this town. The FS Midlands team conducted initial descriptive epidemiology of cases notified to UKHSA from January 2010.

### Descriptive epidemiology and network diagrams

Cases were defined as confirmed or probable. A confirmed case had culture-confirmed TB with a WGS result within an existing TB WGS cluster in Town X and an epidemiological link to any other WGS cluster case, notified since January 2010. A probable case had laboratory-confirmed TB with clinical compatible illness or clinically diagnosed TB, with an epidemiological link to a confirmed case but no WGS result, notified since January 2010. Case data were extracted from the National Tuberculosis Surveillance System and the HPT case management system (HPZone), supplemented with local TB service intelligence and WGS results provided by UKHSA’s Field Services.

Twenty-nine individuals met the case definition (24 confirmed and 5 probable). Of recently notified cases (2020 to 2022; 3 confirmed and 4 probable), 100% were born outside of the United Kingdom and had experienced homelessness. The three recently confirmed cases belonged to three of the five WGS clusters in Town X, indicating continued transmission within these clusters.

A population at risk for TB transmission – PEH – was identified through epidemiological investigations. Therefore, it was agreed by the incident management team (IMT) to conduct a targeted one-day TB-screening event for this group.

### Details of the TB-screening event for PEH in town X

Following the identification of the homeless outreach centre in the descriptive epidemiology, this voluntary and community sector organization (VCSO) was included in planning the multi-agency TB-screening event. The event was held at a local church less than 50 meters from the location of the VCSO to accommodate numerous health and social service providers and a mobile TB-screening van on site. The VCSO led the promotion of the event amongst its service users. Attendees underwent an initial TB assessment by the local TB service and were offered an interferon gamma release assay (IGRA) and a chest x-ray. A paper TB-screening questionnaire was used to record information for attendees, including demographics (age, sex, ethnicity, and country of birth), history of Bacille Calmette-Guerin (BCG) vaccination, TB symptoms, TB risk factors (e.g. travel outside of the United Kingdom, contact with someone with TB), and on-the-day investigations (IGRA, chest x-ray). Remote translation services were available to support the screening event and its evaluation for PEH whose first language was not English, where clinical staff were not conversant in PEH attendees’ language of choice. The local authority provided a packed lunch and self-care package (toiletries) for PEH attendees who underwent screening.

Wider health and social services were also invited to the screening event to provide support and advice to attendees as agreed within our IMTs to promote wider health promotion activities. The services included a community NHS Trust vaccination team, substance misuse support services, smoking cessation advisors, a housing association, integrated sexual health service, specialist neighbourhood practitioners, and a sexual health charity.

### Real-time evaluation and its use in interventions designed for PEH

Real-time evaluation (RTE) is designed to provide immediate (real-time) feedback to those planning or implementing a project or programme, so that they can make improvements during the event and for future events. RTEs are normally associated with emergency response or humanitarian interventions [11], but this evaluation approach can be applied to other scenarios.

A systematic review of screening programmes for active TB amongst PEH in Organization for Economic Co-operation and Development (OECD) countries identified loss to follow-up before diagnosis in multiple studies [12] demonstrating the value of concurrent testing with immediate results as performed in this screening event. None of the included studies explicitly include reference to participant evaluation in their respective studies either during or after screening [12]. The Medical Research Council’s (MRC) latest guidance on designing and evaluating complex health interventions states the importance of meaningful engagement with stakeholders including service users at every stage of design and delivery of interventions to maximize their impact and effectiveness [13]. The Local Government Association’s (LGA) briefing paper reflecting on lessons learned from the COVID-19 pandemic and the needs of local public health from UKHSA states the importance of locally driven processes and responses compared to ‘top-down’ prescribed systems to build health protection capabilities of the future [14].

There are numerous studies utilizing mixed methods evaluation for interventions designed for PEH. Whilst many include service users in evaluation [15, 16], several do not [17–19]. Post-intervention process evaluation has the benefit of directed enquiry, based on initial quantitative findings in sequential mixed-method studies. However, loss to follow-up amongst PEH within health settings could challenge this specific mixed-methods approach for this population [14]. RTE provides an additional opportunity to gather immediate participatory insights into health interventions for this group that may otherwise be overlooked, which is amenable to concurrent mixed-method study design [20]. A recent study demonstrates a framework for using RTE within a targeted chlamydia-screening programme, resulting in a number of impactful changes to the programme that the authors believe improved its effectiveness [21].

### Study rationale

Following epidemiological investigations, PEH in Town X were identified as the population at risk for TB transmission. Engagement of PEH with our targeted one-day TB-screening event and subsequently with healthcare services for diagnosis and treatment would help prevent further person-to-person transmission of TB. However, the uptake or use of healthcare services by PEH could be impacted by numerous factors. These include difficulties in navigating and accessing healthcare services, engagement issues related to distrust in institutions or healthcare providers, disenfranchisement or stigmatization, and ‘chaotic’ lifestyles where health and care are not immediate priorities [22]. Additionally, attitudinal issues from service providers resulting from a combination of stigmatization and a lack of confidence or understanding of working with PEH may impact the uptake or use of healthcare services by PEH [22].

Understanding the experiences and opinions of PEH in the context of targeted public health interventions such as TB screening is vital in shaping future public health interventions and, in turn, improving health outcomes for this group. However, there is no published literature that utilizes real-time evaluation within the context of targeted screening for tuberculosis amongst PEH.

Therefore, our aim was to explore the suitability of RTE as a method of evaluation of a TB-screening event for PEH.

Our objectives were as follows:

1. Organize a TB-screening event for PEH in Town X.
2. Conduct an RTE of our targeted TB-screening event through free-text structured interviews with consenting PEH attendees of our targeted TB-screening event.
3. To assess the level of understanding of TB, the screening process, and result notification in consenting PEH attendees of our targeted TB-screening event.
4. Identify barriers and facilitators to engagement with TB screening amongst consenting PEH attendees of our targeted TB-screening event.
5. Identify additional support services or health promotion partners that would be beneficial for future TB-screening events targeted at PEH.

## Methods

Our RTE involved one-to-one free-text structured interviews with our target users (PEH) and was performed during the multi-agency screening event. PEH are largely unexplored within medical research, so we adopted a free-text structured interview approach to ensure we could capture a range of perspectives. All participants were invited to complete the RTE after their TB-screening assessment and after interacting with any wider health and social services present. Demographic characteristics were captured for all screening attendees but not for those additionally involved in RTE. Local public health intelligence was sought to clarify the numbers and natures of PEH in Town X. Participants were consented to participate in the RTE immediately after screening. Our RTE interviews were held in a shared clinical area immediately after screening to maximize engagement with participants. A copy of our data collection tool for these interviews can be found in Supplementary File 1. Questions covered understanding of TB; how individuals found out about the screening event, concerns about the screening event, thoughts on the explanation of the IGRA and chest x-ray, comfortability with next steps, any suggestions for changes to the day that could have encouraged participation, helpfulness of wider services available on the day, thoughts on whether wider services could have been provided in a better way, and suggestions for any other services that attendees felt should have been present at the screening event. Interviews were performed by members across a multi-professional team and handwritten forms were manually transcribed into Microsoft Excel to perform thematic analysis. Codes were assigned to free-text responses that were then developed into summary themes for each of the key questions within the interview.

### Ethics approval

Ethics approval was not required as the data were used by the organizations involved to conduct communicable disease outbreak investigations and RTE formed part of our service evaluation of this intervention. All data were collected within statutory approvals granted to UKHSA for public health disease surveillance and control. Information was held securely and in accordance with the Data Protection Act 2018, GDPR, and Caldicott guidelines.

## Results

Twenty-eight individuals attended the screening event in March 2023, and 54% of them (n=15) participated in our RTE.

### Demographics of screening attendees

Sixty-four percent of attendees were male (18/28). The age of attendees ranged from 23 to 57 years, with a median age of 42 years. Ninety-three percent of attendees (26/28) stated they were registered with a GP. Forty-six percent of attendees stated they were born in the United Kingdom (13/28), whilst the remainder were either born in Poland, Lithuania, or Latvia. Whilst the primary language cited by most attendees was English (15/28, 54%), nearly half of attendees had a primary language that was not English. Polish, Latvian, Lithuanian, and Russian were the other primary languages reported by attendees. The majority of attendees were unemployed (18/28, 64%). Eighteen (64%) provided some address details. Of these, 9 (50%) cited either a local hotel, our VCSO, or a temporary accommodation provider as their residential address. Ten attendees did not provide an address (36%).

### Real-time evaluation (RTE)

#### Understanding of TB

Of the fifteen participants, five (33%) could not describe a key symptom or consequence of tuberculosis, four (27%) demonstrated an understanding of the long-term implications of TB, five (33%) described typical symptoms or clinical presentations that result from tuberculosis, and one (7%) participant’s response could not be assigned to the three themes.

#### Effectiveness of promotion/awareness of screening event

All participants stated they were made aware of the screening event on the same day they attended the event. 5 participants did not clearly state how they found out about the event. Of the remaining 10 participants, most had learned about the event whilst attending the VCSO; however, both the housing association that was included as one of our wider health and social services and a mental health event running on the same day were also mentioned as sources of information on the screening event.

#### Communication about TB-screening tests and results

Most participants (14/15) were satisfied with the explanation for screening tests and how results would be shared with them. One participant was dissatisfied with how the TB-screening tests were explained to them, and another was unclear on how results would be communicated to them. For these participants, we consulted the TB nurses to address these identified concerns at the time of the event.

#### Suggestions to improve TB-screening services provided

Most participants were satisfied with how the event was delivered; however, there were reports of people being scared about the stigma surrounding drug use and not understanding the purpose of screening. Participants mentioned the importance of a positive welcome and involving PEH in organizing/delivering future events. One suggested coordination with another large homeless charity that provide evening meals.

#### Suggestions to improve wider services provided

Most participants were satisfied with the services provided, but some mentioned service providers communicating in English as a key barrier, with lack of trust in using telephone translation services available and a preference for trusted individuals as translators. Two participants mentioned other drug users they knew were afraid of attending the event. One mentioned their partner being a person who injects drugs who was concerned about the ability to provide a blood sample due to challenging veins.

#### Suggestions for further support services

Most participants did not have suggestions for further wider health and social services that could be worthwhile to include in future screening events. A stall focused on dentistry care and check-ups was suggested as an additional service. Key themes emerging from this part of the RTE included it being overwhelming to have so many staff and services present, the importance of trusted individuals to help on the day, and the presence of voluntary sector services to discuss volunteering opportunities.

#### Concerns before attending the multi-agency TB-screening event

The majority of participants (13/15) had no concerns before attending screening. Concerns identified included unease ahead of attending the screening event before arriving due to allergies and discomfort at the provision of wider health promotion services.

### Screening results

Twenty-four screening attendees had an IGRA test (86%), and 26 had a chest x-ray (93%). Two attendees had symptoms suggestive of TB, so a sputum sample was taken for each attendee. All results were negative for latent or active TB.

## Discussion

Real-time evaluations were first used in the 1990s in response to increasing humanitarian crises, where the United Nations High Commissioner for Refugees (UNHCR) required a means to rapidly evaluate the effectiveness and impact of humanitarian responses to inform immediate action [23], and have scarcely been used outside of this context. This study is the first of its kind to utilize real-time evaluation in the context of targeted screening for tuberculosis amongst PEH.

Twenty-eight persons attended our targeted TB-screening event in Town X. Nineteen attendees provided either no address or a temporary accommodation provider address. Assuming those not declaring an address had no address to provide, we hypothesize that these 19 attendees would be legally defined as PEH. We hypothesize that most of the remaining participants would also meet the legal definition of homelessness based on their interaction with our VCSO – a local homeless outreach centre.

Local public health intelligence suggests in March 2023 there were a total of 23 rough sleepers in Town X. However, we were unable to formulate screening uptake rates for rough sleepers as these data do not encapsulate broader forms of homelessness.

Whilst rates of GP registration amongst attendees were high (93%), probing consideration of the representativeness of our sample, these high levels of registration are consistent with national rates of registration (97%) [24].

Efforts to understand the most effective health communication methods for PEH have demonstrated the importance of trusted messengers, alongside verbal, face-to-face engagement [25–27]. Participatory development of a digital health communication campaign for COVID-19 with PEH suggested easily accessible, multilingual, discrimination sensitive, clear, and simple communication methods also help reach PEH [28]. A US qualitative study with PEH additionally suggested that PEH seek information from multiple sources to determine the trustworthiness of messages [29].

The promotion of our targeted TB-screening event for PEH in Town X was led by a local homeless outreach centre (our VCSO) through verbal, face-to-face communication. A broader communication strategy (through a targeted media campaign amongst numerous health and social services including leaflets and posters) was dissuaded in our IMT discussions as there was a concern that these efforts could inadvertently detract engagement amongst PEH.

However, RTE suggested that our nuanced promotion strategy through the local VCSO did not attract PEH who were informed of the event in advance of the screening day as all participants stated they were made aware of the screening event on the same day they attended the event. It is unclear whether this is because our RTE findings were a mismatch with the local VCSO’s engagement with PEH or if potential attendees were informed but chose not to attend. Considering the importance demonstrated by multiple communication methods for PEH being used to verify information and improve trust [29], a broader communication strategy may have been worthwhile.

RTE provided a voice for PEH in Town X to share their perceptions on how to best align healthcare services for their specific needs. A positive welcome and explanation of tests and how results would be shared were facilitators to engagement with our screening event. However, with 46% of attendees not participating in the RTE, attrition bias is worth considering. Whilst we involved our VCSO in promoting our event, their presence was limited during our screening event itself. Trusted partners within health delivery are known to be especially important when designing services for PEH [30]. Our local VCSO partner was consulted separately in planning the event but was not involved directly in our IMT meetings. Involving this partner in these meetings could have encouraged this partner to play a broader role in the delivery of our intervention, including on-the-day presence. This may have improved uptake, especially in groups that may mistrust existing healthcare services as identified by our RTE.

Pre-engagement with PEH in Town X could have permitted us to highlight and address any pre-identified barriers or execute facilitating factors to improve TB-screening uptake. These include improving the understanding of TB and the purpose of screening (which ranged from no to some understanding), improving trust in translation services provided, or the possibility to maximize engagement if screening was held in conjunction with popular weekly offers of food by a local charity. However, using RTE, we were able to clarify information about testing and results for attendees where the need was identified through RTE, enabling us to make real-time modifications to our TB-screening event and, subsequently, engagement with screening attendees who may have been lost to follow-up.

Whilst the wider services provided were well-received by RTE participants, achieving a balance between overwhelming attendees and providing the most useful services to PEH should be considered when organizing future TB-screening events targeted at PEH.

## Conclusion

We found that RTE was a suitable method of evaluation of a TB-screening event for PEH in Town X. RTE provided us insights into understanding of TB, screening, and results notification processes amongst PEH in Town X and enabled us to identify barriers and facilitators to attending TB screening by PEH in Town X and identify additional support services or health promotion partners that would be beneficial for future TB-screening events targeted at PEH.

Whilst prior engagement with PEH in Town X would have been beneficial in improving TB-screening uptake, RTE enabled us to obtain immediate feedback from PEH who may have been otherwise lost to follow-up. This enabled modification of the screening event in real time, which a conventional longer-term evaluation would not have enabled us to do. We hope through considering factors presented in this article in the planning and delivery of TB-screening events for PEH in the future, including incorporation of RTE, public health teams will achieve high levels of engagement with TB screening and treatment to subsequently improve health outcomes for PEH – a group more vulnerable to TB transmission and poorer TB outcomes.

## Supporting information

Dave et al. supplementary material Dave et al. supplementary material

## Data Availability

Data are incorporated into the article and material contained within. Individual-level data are confidential and cannot be shared.

## Supplementary material

For supplementary material accompanying this paper visit http://doi.org/10.1017/S0950268824000402. click here to view supplementary material
