# Supplementary material for: Real-time evaluation of a multi-agency TB-screening event for persons experiencing homelessness in a town with a low incidence of TB in England
Source: Epidemiol Infect. 2024 Apr 1;152:e73. doi: 10.1017/S0950268824000402 (PMC11094373; doi:10.1017/S0950268824000402)
Supplement: Dave et al. supplementary material [file S0950268824000402sup001.docx]

**TB Screening RTE Data Collection Tool**

**What do you understand about TB and its risk?**

**How did you find out about the screening/health promotion event?**

**Did you have any concerns /difficulties before attending the screening?**

**Do you feel the CXR /blood test were explained to you well?**

**Is there anything we could have done differently to encourage you or your friends to participate?**

**Do you feel comfortable with the next steps?**

**Other services available**

**Which of the wider services / facilities available were helpful to you? (Prompt – service list:**

- Vaccination team
- Substance misuse services
- Smoking cessation advice
- Housing, hostel, and employment advice
- Sexual health service
- Specialist Neighbourhood Practitioners – Engagement team
- Sexual Health Charity

**Of the wider services/facilities available do you wish any of these could have been provided in a better way?**

**Are there any other services that you would have liked to have been here today?**
